# ATP synthase inhibition, an overlooked confounding factor in the mitochondrial stress test

**DOI:** 10.1371/journal.pone.0328256

**Published:** 2025-07-17

**Authors:** Jesse Corbin, Eric A. Lehoux, Isabelle Catelas

**Affiliations:** 1 Department of Mechanical Engineering, Faculty of Engineering, University of Ottawa, Ottawa, Ontario, Canada; 2 Department of Surgery, Faculty of Medicine, University of Ottawa, Ottawa, Ontario, Canada; 3 Department of Biochemistry, Microbiology, and Immunology, Faculty of Medicine, University of Ottawa, Ottawa, Ontario, Canada; Rutgers: Rutgers The State University of New Jersey, UNITED STATES OF AMERICA

## Abstract

The mitochondrial stress test, a widely used procedure to study energy metabolism using extracellular flux analysis, involves the inhibition of ATP synthase (a.k.a. complex V [CV]). This inhibition was recently shown to cause a glycolysis-dependent underestimation of two key mitochondrial respiration parameters, *maximal respiration* (*MR*) and *spare respiratory capacity* (*SRC*), in tumor cells. However, it is unknown if test substances (toxins, drugs, signaling molecules, etc.), especially those affecting glycolysis, can impact the underestimation of *MR* and *SRC* caused by CV inhibition and thereby produce potentially erroneous results. The objective of the present study was to determine if the inhibition of CV in the mitochondrial stress test can act as a confounding factor when measuring *MR* and *SRC* in intact non-tumor cells exposed to exemplificatory test substances that affect energy metabolism: Ni^2+^ and lipopolysaccharides (LPS). Murine bone marrow-derived macrophages were exposed to Ni^2+^ (0–72 ppm) or LPS (0 or 1 µg/mL), and oxygen consumption rates were measured by extracellular flux analysis using the mitochondrial stress test, with and without CV inhibition. Results showed that CV inhibition masked the decrease in *MR* induced by Ni^2+^ or LPS. It also caused the lack of a statistically significant effect of Ni^2+^ on *SRC* to present as an increase of *SRC*, and the LPS-induced decrease of *SRC* to be masked. Results further showed that these erroneous results arose because exposure to Ni^2+^ or LPS reduced the underestimation of *MR* and *SRC* caused by CV inhibition. This phenomenon was associated with increased glycolytic flux. Finally, results confirmed that underestimation of *MR* and *SRC* induced by CV inhibition can occur in non-tumor cells. In conclusion, the present study demonstrates that CV inhibition can act as a confounding factor leading to erroneous conclusions when the mitochondrial stress test is used with intact cells exposed to test substances.

## Introduction

The recent proliferation of studies on energy metabolism has led to a growing appreciation of mitochondria as intracellular signaling hubs in health and disease [1–3]. This proliferation has been driven, in part, by the advent of phosphorescent/fluorescent probe-based extracellular flux analysis (EFA), which has revolutionized the field of bioenergetics. More specifically, the widely used EFA-based mitochondrial stress test [4,5] has facilitated the study of mitochondrial function and its alteration in response to the exposure of cells to drugs, toxins, signaling molecules, or other test substances [6–8]. This stress test, described in more detail below, involves the inhibition of ATP synthase, i.e., complex V (CV) of the mitochondrial electron transport chain (ETC). In a recent study, Ruas *et al.* [9] demonstrated that this inhibition can lead to an underestimation of the maximal capacity of the mitochondrial ETC system in intact (i.e., non-permeabilized) tumor cells. The present study expands upon this research to determine if CV inhibition can act as a confounding factor when using the mitochondrial stress test to analyze the effects of test substances on the energy metabolism of intact non-tumor cells.

The mitochondrial stress test analyzes mitochondrial function by measuring oxygen consumption rates (OCR) before and after sequential additions of the following drugs: a CV inhibitor (oligomycin), an uncoupler (e.g., trifluoromethoxy carbonylcyanide phenylhydrazone [FCCP]), and a mixture of ETC complex I and III inhibitors (e.g., rotenone and antimycin A, respectively). This test provides measurements of key respiration parameters: *basal* (or *resting*) *respiration*, *ATP-linked respiration*, *maximal respiration* (*MR*), *spare respiratory capacity* (*SRC*; a measure of the ability of cells to respond to an increase in energy demand), *proton leak-linked respiration,* and *non-mitochondrial oxygen consumption* [4].

As previously mentioned, it was recently shown that the inhibition of CV in the mitochondrial stress test can cause an underestimation of *MR,* and consequently *SRC,* in intact tumor cells [9]. This underestimation was independent of the source of the inhibitor (oligomycin from MilliporeSigma or Cayman Chemical), its isomeric composition (mixture of oligomycin A, B, and C forms, or pure A form), and the inhibiting agent (oligomycin or citreoviridin) [9]. Underestimation was also unaffected by replacing the uncoupler carbonyl cyanide 3-chlorophenylhydrazone (CCCP) with FCCP [9]. Although the precise mechanism(s) remains unclear, the underestimation of *MR* and *SRC* has been shown to depend on glycolysis and may involve the maintenance of intracellular ATP concentration by inhibition of ATP hydrolysis through the ATPase activity of CV [10] – oligomycin inhibits both the ATP synthase and ATPase activity of CV.

More recently, data published by Rossi *et al.* [11] indicated that the underestimation of *MR* and *SRC* caused by the inhibition of CV can also occur in non-tumor cells (primary neurons). However, it remains unknown if drugs, toxins, signaling molecules, or other test substances, especially those affecting glycolysis, can impact the underestimation of *MR* and *SRC* caused by CV inhibition. Should they do so, CV inhibition would act as a confounding factor when the mitochondrial stress test is applied to intact cells, thus leading to potentially erroneous conclusions regarding the effects of test substances on mitochondrial function. Therefore, the objective of the present study was to determine if the inhibition of CV in the mitochondrial stress test can act as a confounding factor when measuring *MR* and *SRC* in intact non-tumor cells exposed to exemplificatory test substances that affect energy metabolism: Ni^2+^ and lipopolysaccharides (LPS).

## Materials and methods

### Nickel ions and lipopolysaccharides

Stock solutions of Ni^2+^ were prepared fresh, as previously described [12]. Briefly, NiCl_2_·6H_2_O (99.999% purity; MilliporeSigma, St. Louis, MO; catalog no. 203866) was dissolved in cell-culture-grade water (Cytiva, Marlborough, MA), and the solutions were sterilized by filtration through 0.2-μm pore-size cellulose acetate syringe filters (VWR, Radnor, PA). Stock solutions of gamma-radiation-sterilized LPS from *E. coli* O55:B5 (MilliporeSigma) were prepared in Hank’s balanced salt solution without phenol red (Wisent, St-Jean Baptiste, QC), aliquoted, and stored at −20°C until use.

### Animals

All procedures were approved by the University of Ottawa Animal Care Committee (protocols ME-3363 and ME-4364). The University of Ottawa animal care and use program meets the Canadian Council on Animal Care (CCAC) guidelines and is licensed under the Province of Ontario Animals for Research Act. Wild-type female C57BL/6J mice (The Jackson Laboratory, Bar Harbor, ME) were housed at the Animal Care Facility of the University of Ottawa, a specific-pathogen-free (SPF) facility. Female mice were used exclusively (for housing considerations) since sex differences are unlikely to affect the fundamental mechanisms under study, although they might affect the magnitude of the observed responses. Mice (2–5 per cage) were housed in individually ventilated cages (Sealsafe Plus GM500; Techniplast, West Chester, PA) with 6-mm size corncob bedding (Envigo RMS, Indianapolis, IN), cotton fiber-based nesting material (Ancare, Bellmore, NY), and a shreddable refuge hut (Ketchum, Brockville, ON). The animals were maintained at 22°C with a relative humidity of 40% under a 12 h light:12 h dark photoperiod with *ad libitum* access to food (Teklad Global 18% Protein Rodent Diet; Envigo RMS) and water (purified by reverse osmosis and acidified to pH 2.5–3.0 with hydrochloric acid). The mice (n = 11; 9 ± 1 [range 6–10] weeks old; body mass: 18.9 ± 0.8 [range 15–22] g) were euthanized between 8h00 and 15h00 by CO_2_ gas asphyxiation followed by cervical dislocation. Euthanized mice were soaked with 70% (v/v) ethanol immediately prior to dissection.

### Bone marrow-derived macrophages

Bone marrow cells were harvested from tibiae and femora isolated from euthanized mice, and prepared as previously described [12], except that Dulbecco’s modified Eagle medium (DMEM)-based complete growth medium (CGM) was used (DMEM [Wisent], 8% [v/v] heat-inactivated fetal bovine serum [FBS] containing < 0.06 EU/mL of endotoxin [MilliporeSigma, catalog no. F1051], and 100 U/mL each of penicillin and streptomycin [Cytiva]). The cells were counted by dye-exclusion hemocytometry with trypan blue (0.04% [w/v] final concentration; MilliporeSigma) using an Improved Neubauer hemocytometer (Hausser Scientific, Horsham, PA), and seeded at ca. 230,000 cells/cm^2^ in polystyrene Petri dishes (Greiner Bio-One, Monroe, NC) coated with recombinant macrophage colony-stimulating factor (M-CSF, 0.85 µg/cm^2^; R&D systems, Minneapolis, MN), as per Sadh *et al.* [13]. The seeded cells were incubated for 6 days in CGM supplemented with β-mercaptoethanol (55 µM; ThermoFisher Scientific, Rockford, IL), under cell culture conditions (37°C, humidified atmosphere of 95% air and 5% CO_2_). On day 3, pre-warmed (37°C) CGM supplemented with ß-mercaptoethanol (55 µM) and M-CSF (5 ng/mL) was added to the culture supernatants (0.4:1.0 v:v). At the end of the 6-day incubation, non-adherent cells were removed by rinsing with pre-warmed (37°C) CGM and the BMDM were detached using a 2-cm blade polyethylene cell lifter (Fisher Scientific, Waltham, MA). The bone marrow-derived macrophages (BMDM) were collected by centrifugation (300 × *g* for 10 min) and resuspended at 4.0 × 10^5^ cells/mL in pre-warmed (37°C) CGM freshly supplemented with M-CSF (5 ng/mL).

### Extracellular flux analysis

EFA was performed using an extracellular flux analyzer (Seahorse XFe96; Agilent Technologies, Santa Clara, CA). Briefly, the cartridge sensors (Seahorse XFe96/XF Pro Extracellular Flux Assay Kit; Agilent Technologies) were hydrated overnight at 37°C in a calibration solution (XF Calibrant Solution; Agilent Technologies), as per the manufacturer’s 2020 instructions. Specially-designed polystyrene tissue culture-treated 96-well microplates with a clear flat bottom (Seahorse XFe96/XF Pro Cell Culture Microplate; Agilent Technologies) were then seeded with 80 µL of cell suspension (above) to produce a cell density of ca. 300,000 cells/cm^2^, left undisturbed at room temperature for 1 h to reduce edge effects [14], and incubated for 16 h under cell culture conditions to allow cell attachment/recovery. At the end of the incubation, the culture supernatants were replaced with growth medium containing Ni^2+^ (6–72 ppm), LPS (1 µg/mL), or CGM (negative control). The cells were then incubated for 6 h under cell culture conditions.

### Mitochondrial stress test

At the end of the 6-h incubation, the adherent cells were washed (as per Agilent Technologies’ 2023 instructions) and incubated for 45 min at 37°C under atmospheric O_2_ and CO_2_ concentrations in OCR medium (DMEM without sodium bicarbonate, L-glutamine, D-glucose, Na-pyruvate, and phenol red [Wisent] supplemented with 4 mM L-glutamine [Wisent], 4.5 g/L D-glucose [Wisent], 1.25 mM Na-pyruvate [MilliporeSigma], and 5 mM 4-(2-hydroxyethyl)-1-piperazineethanesulfonic acid [HEPES; MilliporeSigma], pH 7.35 ± 0.05 at 37°C). Oxygen consumption rates (OCR) were measured to assess *basal respiration*, followed by *ATP production-dependent respiration*, *MR*, and *non-mitochondrial oxygen consumption*, after sequential injections of oligomycin A (Cayman Chemical, Ann Arbor, MI, catalog no. 11342), trifluoromethoxy carbonylcyanide phenylhydrazone (FCCP; Cayman Chemical), and rotenone together with antimycin A (MilliporeSigma) to final concentrations of 1, 2, 0.5, and 0.5 µM, respectively. To determine *MR* and *SRC* in the absence of CV inhibition, the injection of oligomycin A was replaced with an injection of OCR medium, and FCCP was injected to a final concentration of 3 µM in half of the samples and controls from each experiment. FCCP was titrated in both the presence and absence of oligomycin A (S1 Fig). The observation that a higher concentration of FCCP is required to achieve *MR* in the absence of oligomycin A is in agreement with previous results, and an explanation for this phenomenon has been proposed [9]. OCR measurements were performed, at 6-min intervals, thrice before the first injection and thrice, thrice, and twice, after each of the sequential injections. The presence of trace ethanol and/or dimethyl sulfoxide in the injected solutions had no detectable effect on OCR, except possibly in the vehicle of rotenone and antimycin A, which may have caused a small increase in OCR (S2 Fig). The following OCR measurements (i.e., time points) were selected for data analysis based on empirical considerations [4]: the last two measurements pre- and post-oligomycin or OCR medium injection, the first measurement post-FCCP injection, and both measurements post-rotenone/antimycin A injection. All OCR measurements were corrected for the OCR of cell-free wells containing only OCR medium and normalized to cell number (see below).

*Basal respiration* was calculated by subtracting OCR after rotenone and antimycin A injection (i.e., *non-mitochondrial oxygen consumption*) from OCR pre-injections. *ATP-linked respiration* was calculated by subtracting OCR after oligomycin A injection from OCR pre-injections. *MR* was calculated by subtracting OCR post rotenone and antimycin A injection from OCR after FCCP injection. *SRC* was calculated by subtracting *basal respiration* from OCR after FCCP injection. *Proton leak-linked respiration*, i.e., mitochondrial respiration that is not coupled to ATP production, was calculated by subtracting OCR after rotenone and antimycin A injection from OCR after oligomycin A injection.

### Glycolysis stress test

The improved glycolysis stress test used was based on Mookerjee *et al.* [15]. At the end of the 6-h incubation, the adherent cells were washed (as above) and incubated for 45 min at 37°C under atmospheric O_2_ and CO_2_ concentrations in base ECAR medium (DMEM without sodium bicarbonate, L-glutamine, D-glucose, Na-pyruvate, and phenol red [Wisent], but supplemented with 4 mM L-glutamine [Wisent] and 5 mM HEPES [MilliporeSigma], pH 7.35 ± 0.05 at 37°C). Immediately before use, the base medium was supplemented with carbonic anhydrase (500 U/mL final; Worthington Biochemical, Lakewood, NJ), as previously described [16]. Extracellular acidification rates (ECAR) were measured to assess *basal acidification* (i.e., acidification in the absence of exogenous glucose), followed by *basal glycolysis*, *ATP demand-limited glycolysis*, and *maximal glycolytic capacity*, after sequential injections of D-glucose, rotenone together with antimycin A, and monensin (an ionophore used to increase the rate of ATP hydrolysis by the Na^+^/K^+^-ATPase; Cayman Chemical) together with FCCP, to final concentrations of 10 mM, 0.5 µM, 0.5 µM, 10 µM, and 3 µM, respectively. 2-Deoxy-D-glucose (2-DG; Cayman Chemical; a non-metabolizable analog of D-glucose that inhibits glycolysis primarily through competitive inhibition of phosphoglucoisomerase) was injected last (50 mM post injection) to confirm that the measured ECAR were glucose dependent. ECAR measurements were performed at 6-min intervals, twice before the first injection and thrice after each subsequent injection. The following ECAR measurements (i.e., time points) were selected for data analysis based on empirical considerations: the two measurements before glucose injection, all three measurements post-glucose, -rotenone/antimycin A, −2-DG injections, and the last two measurements post-FCCP/monensin injection. All ECAR measurements were corrected for pH changes in cell-free wells containing only ECAR medium, and normalized to cell number (see below).

Proton efflux rates (PER) were computed using Wave Desktop software v.2.6.3 (Agilent Technologies). The buffering factor (2.4 ± 0.0 mmol H^+^/L/pH unit) used in the PER calculations was determined experimentally as per Agilent Technologies [17]. A CO_2_ contribution factor (CCF) of 0.61 (Agilent Technologies) was used. The CCF was assumed to be unaffected by the presence of Ni^2+^ – our group previously demonstrated that CCF was not significantly affected by Co^2+^ at a concentration of 24 ppm [16].

*Basal glycolysis* was calculated by subtracting glycolytic PER before glucose injection from glycolytic PER after glucose injection. *ATP-demand limited glycolysis* was calculated by subtracting PER before glucose injection from PER after rotenone and antimycin A injection. *Maximal glycolytic capacity* was calculated by subtracting glycolytic PER before glucose injection from (glycolytic) PER after FCCP and monensin injection. *Glycolytic reserve*, a measure of the ability of cells to respond to increased energy demand, was calculated by subtracting glycolytic PER after glucose injection from (glycolytic) PER after FCCP and monensin injection.

### ATP production rates

ATP production rates were calculated as per Agilent Technologies [18] except that mitochondrial ATP production rates were calculated using *ATP-linked respiration* (OCR) from the mitochondrial stress test, while glycolytic ATP production rates were calculated using *ATP-demand-limited glycolysis* (glycolytic PER) from the glycolysis stress test.

### Cell counting for EFA data normalization

Immediately after extracellular flux analysis, the BMDM were fixed as described by Skehan *et al.* [19]. Briefly, ice-cold trichloroacetic acid (50% [w/v]; Fisher Scientific) was added to the wells of the microplate to a final concentration of 10% (w/v). The microplate was left undisturbed for 5 min, then incubated at 4°C for 1 h. The fixed BMDM were then washed 5 times with ASTM Type III water, air dried, and stored at room temperature for future automated cell counting.

The fixed cells were permeabilized using a detergent solution (Triton X-100 [MilliporeSigma]; 0.3% [v/v] in Dulbecco’s phosphate-buffered saline [DPBS] without Ca^2+^ and Mg^2+^ (Wisent) and their nuclei were stained for 10 min with 4′,6-diamidino-2-phenylindole dihydrochloride (DAPI [MilliporeSigma]; 0.1% [w/v] in DPBS without Ca^2+^ and Mg^2+^). The fixed and stained cells were imaged by automated multichannel fluorescence microscopy (model EVOS FL Auto 2 Cell Imaging System; ThermoFisher Scientific) using a 20 × objective with a 0.40 numerical aperture, and a DAPI filter cube (BP 357/44 nm excitation filter, BP 447/60 nm emission filter). The imaged cells were then automatically counted using Python-language code from Bhattiprolu [20] (incorporating the StarDist object detection model) modified to segment each well image and count the total number of stained nuclei per well. The number of BMDM per well was slightly higher in the presence of Ni^2+^ than in its absence (presumably due to increased retention of attached cells), but similar in the absence or presence of LPS (S3 Fig).

### Statistical analysis

Unless otherwise specified, statistical analysis was performed using GraphPad Prism v10.4.0 for MacOS. Outliers at both the technical and experimental levels were identified using Tukey’s fences implemented in a custom script written in Python v3.10.7. The data were considered to meet the assumptions of normality, and Levene’s test implemented in R v4.4.3 [21] was used to confirm that the assumption of homoscedasticity was met. Statistical methods used to compare the means are described in the figure legends. *p* < 0.05 was considered significant. Effect sizes are presented as Cohen’s *d* with 95% confidence intervals (CI). Unless otherwise specified, data are presented as means ± standard errors of the mean (SEM) of 3 or 4 independent experiments, each performed with cells from a single mouse and sextuplicate samples per condition.

## Results

**Fig 1:**
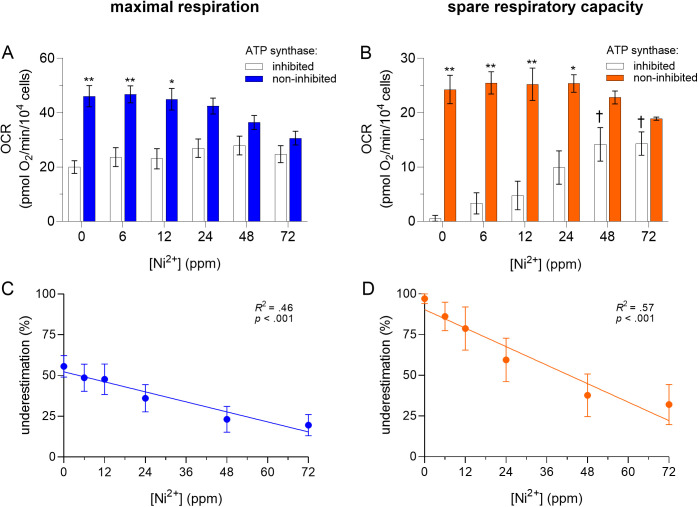
Effects of Ni^2^^+^ on the underestimation of *MR* (A, C) and *SRC* (B, D) in BMDM. Murine bone marrow-derived macrophages (BMDM) were exposed to Ni^2+^ (0–72 ppm) for 6 h, then oxygen consumption rates (OCR) were measured by extracellular flux analysis using the mitochondrial stress test with and without ATP synthase (CV) inhibition using oligomycin A (1 µM). OCR values were normalized to cell number, as determined by automated microscopy. Asterisks (*, **) indicate a significant difference (*p *< 0.05 and *p *< 0.001, respectively) between *maximal respiration* or *spare respiratory capacity* measured with and without CV inhibition at given concentration of Ni^2+^. A dagger (†) indicates a significant difference (*p* < 0.05) between a given condition with CV inhibition and its corresponding negative control (BMDM unexposed to Ni^2+^). The corresponding statistics for conditions without CV inhibition are presented in Fig. 2A, where the same data is presented. A two-way analysis of variance (ANOVA) was performed followed by the Tukey post-hoc test. Linear regressions for *MR*: slope = −0.52 ± 0.12, *R*^2^ = 0.46, *F*(1, 22)=18.7, *p *< 0.001, and *SRC*: slope = −0.95 ± 0.18, *R*^2^ = 0.57, *F*(1, 21)=27.3, *p *< 0.001). An *F* test was used to determine if the slopes were significantly different than zero. Data are presented as means ± SEM of 4 independent experiments, each performed with sextuplicate samples.

To verify that the inhibition of CV can result in the underestimation of *MR* and *SRC* in non-tumor (intact) cells, the mitochondrial stress test was performed with untreated BMDM in the presence and absence of CV inhibition with oligomycin A. Results showed that CV inhibition caused the stress test to underestimate *MR* and *SRC* by 56 ± 7% (*p *< 0.001, *d* = 4.1, 95% CI [1.0, 7.1]; Fig. 1A, 1C; 0 ppm Ni^2+^) and 97 ± 3% (*p *< 0.001, *d* = 6.3, 95% CI [2.1, 10.5]; Fig. 1B, 1D; 0 ppm Ni^2+^), respectively.

**Fig 2:**
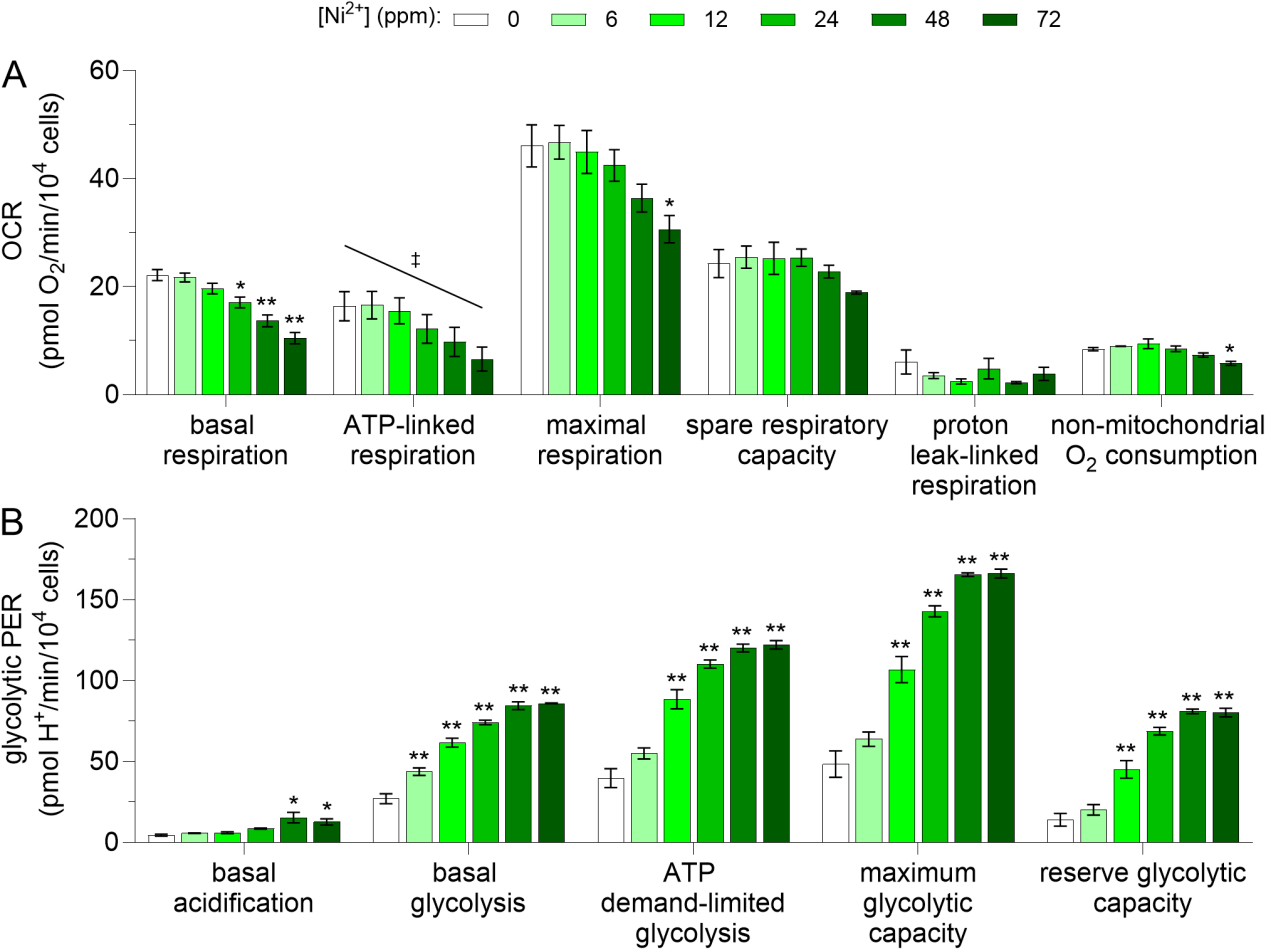
Effects of Ni^2^^+^ on the mitochondrial and glycolytic energy metabolism of BMDM. Murine bone marrow-derived macrophages (BMDM) were exposed to Ni^2+^ (0–72 ppm) for 6 h, then oxygen consumption rates (OCR) **(A)** and glycolytic proton efflux rates (PER) **(B)** were determined by extracellular flux analysis using the mitochondrial and glycolysis stress tests, respectively. The OCR parameters were calculated using data collected with or without ATP synthase [CV] inhibition, as follows. *Basal respiration*: average of data collected with and without CV inhibition. *ATP-linked respiration* and *proton leak-linked respiration*: data collected with CV inhibition. *Maximal respiration* (*MR*), *spare respiratory capacity* (*SRC*), and *non-mitochondrial O*_*2*_ *consumption*: data collected without CV inhibition. Note that the *MR and SRC* data presented in this figure were also presented in Fig 1A and 1B, respectively. Glycolytic PER were calculated as described under *Materials and methods*. Both OCR and PER measurements were normalized to cell number, as determined by automated microscopy. Asterisks (*, **) indicate a significant difference (*p *< 0.05 and *p *< 0.001, respectively) between a given condition and its corresponding negative control (BMDM unexposed to Ni^2+^) (one-way ANOVA followed by Dunnett’s post-hoc test). A double dagger (‡) indicates a significant decrease of OCR (slope = −0.14 ± 0.04, *R*^2^ = 0.40, *F*(1, 22)=15.0, *p *< 0.001) from 0 to 72 ppm Ni^2+^. An *F* test was used to determine if the slope was significantly different than zero. Data are presented as means ± SEM of 4 and 3 independent experiments for OCR and PER, respectively, each performed with sextuplicate samples.

To determine if the underestimation of *MR* and *SRC* caused by CV inhibition can result in erroneous results when analyzing the effects of test substances on these parameters, BMDM were exposed to Ni^2+^. Results showed that CV inhibition masked the effects of Ni^2+^ on *MR*. More specifically, exposure to increasing concentrations of Ni^2+^ did not significantly affect *MR* in the presence of CV inhibition (*p* ≥ 0.84 with all Ni^2+^ concentrations; Fig. 1A), but decreased *MR* in the absence of CV inhibition (33 ± 2% with 72 ppm Ni^2+^, *p *= 0.014, *d* = 2.4, 95% CI [0.1, 4.6]; Fig. 2A). The presence of CV inhibition also caused the effects of Ni^2+^ on *SRC* to differ from those in the absence of CV inhibition. More specifically, exposure to increasing concentrations of Ni^2+^ increased *SRC* in the presence of CV inhibition (from 0.6 ± 0.6 pmol O_2_/min/10^4^ cells without Ni^2+^ to 14 ± 2 pmol O_2_/min/10^4^ cells with 72 ppm Ni^2+^, *p *= 0.005, *d* = 4.4, 95% CI [1.2, 7.6]; Fig. 1B), but had no statistically significant effect on *SRC* in the absence of CV inhibition (*p* = 0.33 at 72 ppm Ni^2+^; Fig. 2A). Interestingly, results also showed that Ni^2+^ induced a concentration-dependent decrease in the underestimation of *MR* and *SRC* caused by CV inhibition (from 56 ± 7% without Ni^2+^ to 20 ± 6% with 72 ppm Ni^2+^, [*R*^2* *^= 0.46, *p *< 0.001; Fig. 1C] and from 97 ± 3% without Ni^2+^ to 32 ± 12% with 72 ppm Ni^2+^ [*R*^2* *^= 0.57, *p *< 0.001; Fig. 1D], respectively).

**Fig 3:**
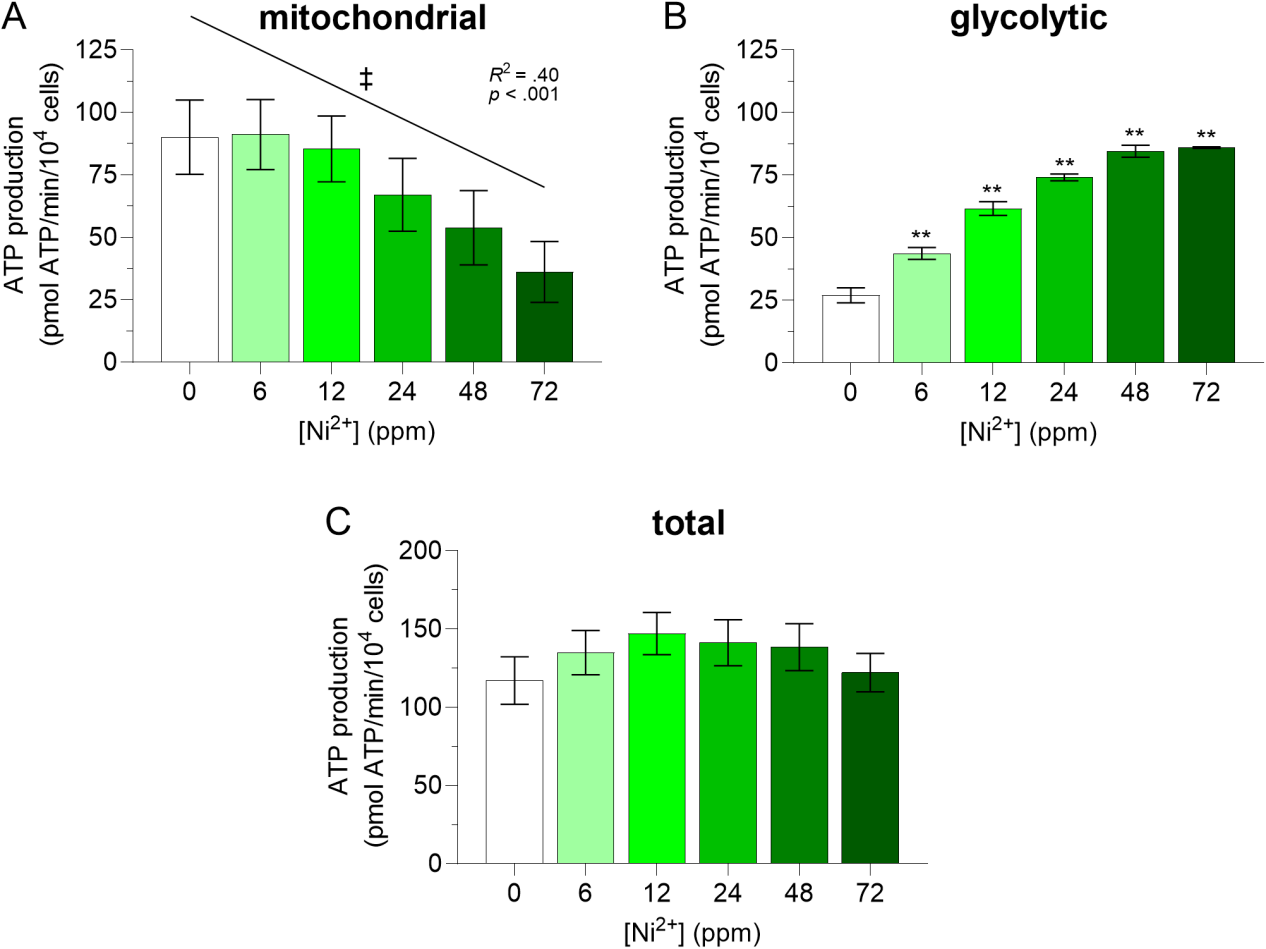
Effects of Ni^2+^ on mitochondrial (A), glycolytic (B), and total (C) ATP production rates in BMDM. Murine bone marrow-derived macrophages (BMDM) were exposed to Ni^2+^ (0–72 ppm) for 6 h, then oxygen consumption rates (OCR) and glycolytic proton efflux rates (PER) were determined by extracellular flux analysis using the mitochondrial and glycolysis stress tests, respectively. Mitochondrial and glycolytic ATP production rates were calculated as described under *Materials and methods*, and normalized to cell number, as determined by automated microscopy. Note that different stress tests and experimental groups (i.e., separate experiments) were used to determine mitochondrial and glycolytic ATP production rates, and therefore to estimate total ATP production rates. A double asterisk (**) indicates a significant difference (*p *< 0.001) between a given condition and its corresponding negative control (BMDM unexposed to Ni^2+^) (one-way ANOVA followed by Dunnett’s post-hoc test). A double dagger (‡) indicates a significant decrease of mitochondrial ATP production rates (slope = −0.79 ± 0.21, *R*^*2*^ = 0.40, *F*(1, 22)=15.0, *p *< 0.001) from 0 to 72 ppm Ni^2+^. An *F* test was used to determine if the slope was significantly different than zero. Data are presented as means ± SEM of 4 and 3 independent experiments for OCR and PER respectively, each performed with sextuplicate samples.

Since the underestimation of *MR* and *SRC* induced by CV inhibition has been attributed to elevated glycolytic activity [10,22], we investigated the effects of Ni^2+^ on both the mitochondrial and glycolytic energy metabolism of BMDM. Results showed that in the absence of Ni^2+^, macrophages relied primarily on mitochondria to meet their energy demand (Figs 2 and 3). Exposure of the BMDM to Ni^2+^ moderately decreased reliance on mitochondrial energy production (Figs 2A and 3A) and increased reliance on glycolytic energy production, in a concentration-dependent way (Figs 2B and 3B). More specifically, all glycolytic PER parameters increased from 3.2 ± 0.5 to 6.7 ± 1.8-fold when Ni^2+^ concentration was increased from 0 to 72 ppm (*p *< 0.001 in all cases, *d* = 11, 95% CI [1.8, 19] to *d* = 11, 95% CI [2.0, 21]; Fig 2B). The effects of Ni^2+^ on mitochondrial PER are shown in S4 Fig.

Since the underestimation of *MR* and *SRC* induced by CV inhibition has been associated with intracellular ATP concentrations [10], ATP production rates were calculated from OCR (*ATP-linked respiration*) and glycolytic PER (*basal glycolysis*) data. Results showed that exposure to Ni^2+^ induced a concentration-dependent decrease in mitochondrial ATP production rates (*p *< 0.001; Fig 3A) and a concentration-dependent increase in glycolytic ATP production rates (up to 3.3 ± 0.3-fold at 72 ppm, *p *< 0.001, *d* = 13, 95% CI [3.7, 21]; Fig 3B). Total intracellular ATP production rates were similar at all Ni^2+^ concentrations (Fig 3C).

**Fig 4:**
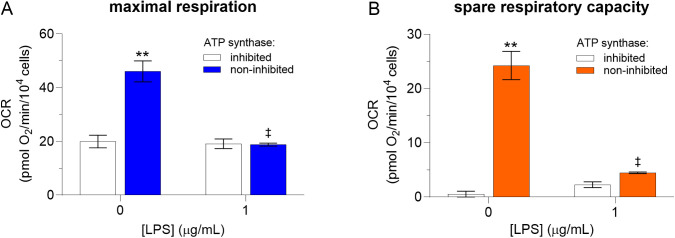
Effects of lipopolysaccharides on *MR* (A) and *SRC* (B) in BMDM. Murine bone marrow-derived macrophages (BMDM) were exposed to lipopolysaccharides (LPS; 0 or 1 µM) for 6 h, then oxygen consumption rates (OCR) were determined by extracellular flux analysis using the mitochondrial stress test with and without ATP synthase (CV) inhibition. OCR values were normalized to cell number, as determined by automated microscopy. A double asterisk (**) and a double dagger (‡) indicate a significant difference (*p *< 0.001) between OCR measured with and without CV inhibition and between OCR measured with cells exposed and unexposed (negative control) to LPS, respectively, under the same CV inhibition condition (two-way ANOVA followed by the Tukey post-hoc test). Data are presented as means ± SEM of 4 and 3 independent experiments for OCR and PER, respectively, each performed with sextuplicate samples. Note that LPS and Ni^2+^ were tested in parallel and thus share the same negative control.

As demonstrated with Ni^2+^, underestimation of *MR* and *SRC* caused by CV inhibition can lead to erroneous results when analyzing the effects of a substance on these parameters. To verify that this phenomenon is not limited to Ni^2+^, we analyzed the effects of another test substance, LPS, on these respiration parameters measured in the presence and absence of CV inhibition. Results showed that CV inhibition masked the effects of LPS on *MR*. More specifically, exposure of the BMDM to LPS did not significantly affect *MR* in the presence of CV inhibition (*p* = 0.99), but decreased *MR* in the absence of CV inhibition (58 ± 3%, *p *< 0.001, *d* = 4.9, 95% CI [1.4, 8.4]; Fig 4A). The presence of CV inhibition also caused the effects of LPS on *SRC* to differ from those in the absence of CV inhibition. More specifically, exposure to LPS had no statistically significant effect on *SRC* in the presence of CV inhibition (*p* = 0.87), but decreased SRC (79 ± 2%, *p *< 0.001, *d* = 4.9, 95% CI [1.0, 8.8]; Fig 4B) in the absence of CV inhibition. Finally, results showed that exposure of the BMDM to LPS reduced the underestimation of *MR* and *SRC* caused by CV inhibition to near nil (−0.3 ± 1.4 pmol O_2_/min/10^4^ cells for *MR*, *p *= 0.999; Fig 4A, and 2.2 ± 1.6 pmol O_2_/min/10^4^ cells for *SRC*, *p *= 0.797; Fig 4B, respectively). The effects of LPS on all OCR, glycolytic PER, and mitochondrial PER parameters are shown in S5 Fig.

**Fig 5:**
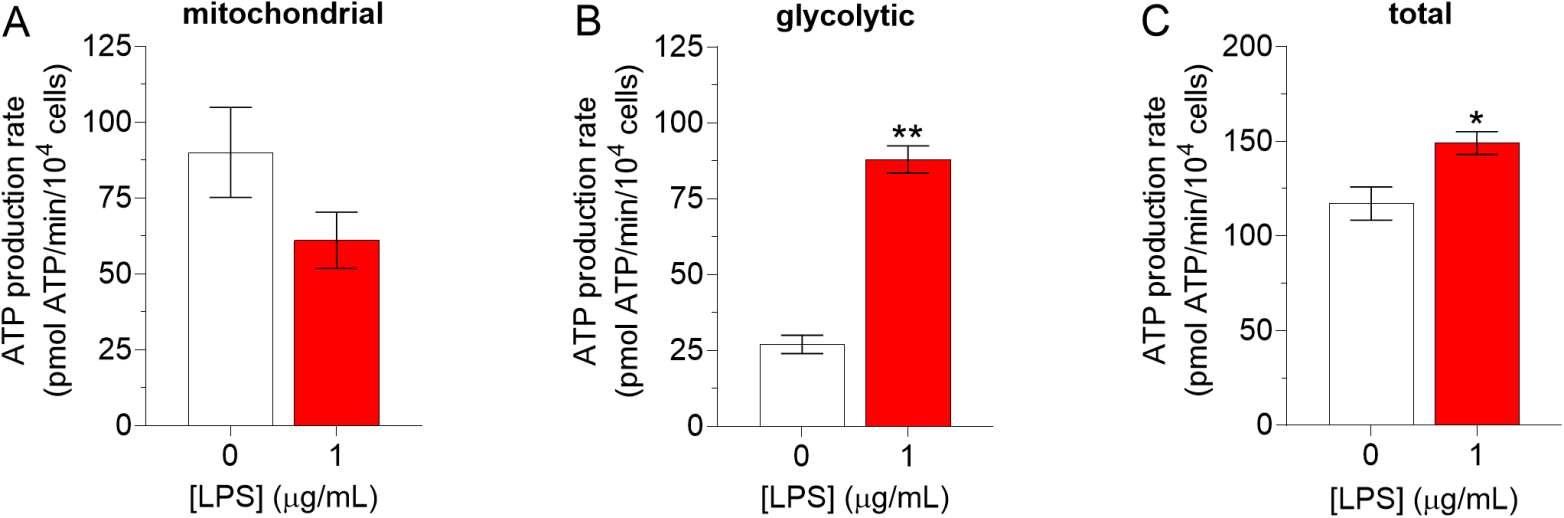
Effects of lipopolysaccharides on mitochondrial (A), glycolytic (B), and total (C) ATP production rates in BMDM. Murine bone marrow-derived macrophages (BMDM) were exposed to lipopolysaccharides (LPS; 0 or 1 µM) for 6 h, then oxygen consumption rates (OCR) and glycolytic proton efflux rates (PER) were determined by extracellular flux analysis using the mitochondrial and glycolysis stress tests, respectively. Mitochondrial and glycolytic ATP production rates were calculated as described under *Materials and methods* and normalized to cell number, as determined by automated microscopy. Note that different stress tests and experimental groups (i.e., separate experiments) were used to determine mitochondrial and glycolytic ATP production rates, and therefore to estimate total ATP production rates. Asterisks (*, **) indicate a significant difference (*p *< 0.05 and *p *< 0.001, respectively) between a given condition and its corresponding negative control (BMDM unexposed to LPS) (Student’s t-tests). Data are presented as means ± SEM of 4 and 3 independent experiments for OCR and PER respectively, each performed with sextuplicate samples. Note that LPS and Ni^2+^were tested in parallel and thus share the same negative control.

Finally, the effects of LPS on ATP production rates were analyzed. Exposure of the BMDM to LPS did not significantly affect mitochondrial ATP production rates (*p *= 0.15; Fig 5A), but increased glycolytic ATP production rates 3.3 ± 0.3-fold (*p *< 0.001, *d* = 9.2, 95% CI [1.5, 17]; Fig 5B) and total ATP production rates 1.3 ± 0.2-fold (*p *= 0.04, *d* = 1.4, 95% CI [−1.1, 4.0]; Fig 5C).

## Discussion

Since their introduction less than two decades ago, EFA-based stress tests have undergone a number of improvements and refinements [15,23–25]. Notwithstanding, these tests remain susceptible to historical assumptions, experimental limitations, and other caveats that can lead to erroneous conclusions [26]. As previously mentioned, it was recently shown that the inhibition of CV in the mitochondrial stress test can induce an underestimation of *MR* and *SRC* in intact tumor-cells [9]. This led to the recommendation that *MR* (and consequently *SRC*) in intact cells be determined in the absence of CV inhibition [9]. However, a survey of the literature reveals that this recommendation has been widely overlooked. In most studies using the mitochondrial stress test with intact cells, *MR* and *SRC* are still measured in the presence of CV inhibition. Several factors may have contributed to this situation, including: the availability of commercial mitochondrial stress-test kits that do not address the potential issue of underestimation of respiration parameters caused by CV inhibition; the extra cost and time associated with performing the mitochondrial stress test both with and without CV inhibition to measure ATP-dependent respiration and *MR*, respectively; and that, until recently [11], underestimation of *MR* and *SRC* in the presence of CV inhibition had only been shown to occur with tumor cells. Furthermore, although measurements of *MR* and *SRC* have proven to be broadly applicable and useful, *MR* and *SRC* are considered non-physiological parameters [26], hence their moderate underestimation may have been assumed to have limited consequences. However, the present study demonstrates that when analyzing the effects of test substances, underestimation of *MR* and *SRC* induced by the inhibition of CV can lead to results that are not only quantitatively incorrect, but also qualitatively incorrect.

Primary (non-tumor) macrophages were used in the present study to confirm that the underestimation of *MR* and *SRC* induced by CV inhibition is not limited to neoplastic or highly glycolytic cells. In addition, energy metabolism has been extensively studied in macrophages, which are archetypal cells for metabolic reprograming (see [27] for a recent review). BMDM prepared from C57BL/6J mice were used because they are a common model for the study of macrophages. The selection of Ni^2+^ as a test substance was based on: 1) our group’s interest in the pathomechanisms induced by corrosion products from metal implants; and 2) our recent demonstration that the exposure of RAW264.7 macrophages to Co^2+^ (another divalent transition metal cation) induced a metabolic shift away from oxidative phosphorylation towards glycolysis [16,28]. LPS, also known as endotoxin, was selected because of its well-established ability to induce a similar metabolic shift in murine BMDM [29].

Results showed that CV inhibition caused an underestimation of *MR* and *SRC* in non-tumor macrophages, thereby confirming that this phenomenon is not limited to neoplastic or highly glycolytic cells. In fact, the underestimation of *SRC* observed with these non-tumor cells (98 ± 3%) is considerably larger than that reported by Ruas *et al.* [9] with tumor cells (20–45%, depending on the cell line). This observation is in general agreement with results published by Rossi *et al.* [11], from which we estimated a negative *SRC* (ca. −45 pmol O_2_/min) in the presence of CV inhibition but a positive *SRC* (ca. 115 pmol O_2_/min) in the absence of CV inhibition, with non-tumor neurons. The magnitude of *MR* and *SRC*, relative to that of the basal respiration measured in the presence of CV inhibition (as per the standard test procedure), is also in general agreement with that of previous studies using murine BMDM (e.g., [6,29–32]), with some exceptions (e.g., [25,33]). The reason for the discrepancy between studies is unclear.

When BMDM exposed to Ni^2+^ were analyzed using the mitochondrial stress test with CV inhibition (as per the standard test procedure), results showed that the effects of Ni^2+^ on *MR* were masked (no statistically significant effect rather than a decrease). Results also showed that the effects of Ni^2+^ on *SRC* differed from those observed without CV inhibition (increase rather than no statistically significant effect). Measurement of *MR* and *SRC* performed in the presence of CV inhibition therefore incorrectly suggested that the exposure of BMDM to Ni^2+^ increased oxidative phosphorylation capacity. This demonstrates that the inhibition of CV in the mitochondrial stress test can act as a confounding factor, which can lead to erroneous conclusions. The observed erroneous results arose because exposure to Ni^2+^ produced a concentration-dependent reduction of the underestimation of *MR* and *SRC* caused by CV inhibition. Results also showed that CV inhibition acted as a confounding factor when BMDM were exposed to LPS – the LPS-induced decrease in *MR* and *SRC* was masked in the presence of CV inhibition. This demonstrates that the phenomenon we report is not limited to a particular test substance and may be widespread. These results emphasize the importance of omitting CV inhibition when the mitochondrial stress test is used to analyze the effects of test substances on intact cells.

Proper determination of both ATP-linked respiration and *MR* may therefore require performing the mitochondrial stress test twice (i.e., with and without CV inhibition) in parallel, as in the present study. However, since 2-DG appears to eliminate the underestimation of *MR* by inhibiting glycolysis, Ruas *et al.* [22] proposed adding 2-DG before CV inhibition as a time- and cost-effective alternative. Unfortunately, this convenient approach prevents the concomitant analysis of OCR and ECAR/PER. Furthermore, under some experimental conditions, 2-DG might affect the results of the mitochondrial stress test. For example, it was recently reported that the exposure of macrophages to 2-DG can impair oxidative phosphorylation and reduce ^13^C-labeled Krebs cycle metabolites and intracellular ATP concentrations [34].

As previously mentioned, underestimation of *MR* and *SRC* caused by the inhibition of CV has been shown to be dependent on glycolysis [10]. Furthermore, intracellular ATP concentration and the intracellular ATP/ADP concentration ratio have been shown to be higher when CV was inhibited [10]. This change in energy status is likely due to the inhibition of ATP hydrolysis via the ATPase activity of CV. A higher intracellular ATP/ADP concentration ratio could result in lower uncoupler-induced *MR* by inhibiting mitochondrial enzymes involved in the reduction of NAD^+^ to NADH, thereby restricting electron transfer to the ETC [10]. Finally, exposing detergent-permeabilized cells to exogenous ATP has been shown to result in the underestimation of *MR* and *SRC* when CV is inhibited, whereas exposure to ADP does not [10]. Together, these results have led to the hypothesis that glycolytic ATP production may exert an inhibitory effect on the metabolism of respiration substrates and cytochrome c oxidase activity [10]. If this hypothesis is correct, a test substance increasing glycolytic ATP production rates would be expected to increase the underestimation of *MR* and *SRC* caused by CV inhibition. However, our results show the opposite: exposure of the BMDM to Ni^2+^ increased glycolytic ATP production rates but decreased the underestimation of *MR* and *SRC* caused by CV inhibition in a dose-dependent way. Exposure to LPS had a similar effect, albeit only a single concentration was tested. Although only LPS significantly increased total ATP production rates, these results raise questions about the mechanism(s) through which the inhibition of CV causes the underestimation of *MR* and *SRC* in the mitochondrial stress test. However, the total ATP production rates we present should be interpreted cautiously because different stress tests and experimental groups were used to estimate mitochondrial and glycolytic ATP production rates. Moreover, the observation that Ni^2+^ and LPS increased glycolytic ATP production rates sufficiently to prevent a decrease in total ATP production rates does not necessarily indicate that intracellular ATP concentration was maintained. The latter and/or the intracellular ATP/ADP concentration ratio could have decreased if the increase in ATP demand exceeded the corresponding increase in glycolytic ATP production. This highlights the limitations of inferring mechanisms based solely on EFA data. Interestingly, exposure to Ni^2+^ has been reported to decrease the intracellular concentration of ATP in a study using L929 murine fibroblasts [35]. Unfortunately, the reported effects of exposure to LPS on intracellular ATP concentration in BMDM are inconsistent [36–38]. Notwithstanding, measurements of intracellular ATP concentration and the intracellular ATP/ADP concentration ratio were considered beyond the scope of the present study, as they relate to the mechanistic aspects of the underestimation of *MR* and *SRC* caused by CV inhibition.

Finally, the main limitations of the present study are the use of a single cell type (murine BMDM) and only two examples of test substances (Ni^2+^ and LPS). Notwithstanding, the inhibition of CV has been shown to induce the underestimation of *MR* and *SRC* in different human cell types [9,11,22], and any change of this underestimation by a test substance will inevitably act as a confounding factor potentially leading to erroneous conclusions, as exemplified in the present study with murine cells exposed to Ni^2+^ or LPS.

## Conclusion

The results of the present study demonstrate that the inhibition of CV can act as a confounding factor leading to erroneous conclusions when the mitochondrial stress test is used to analyze the effects of test substances on the energy metabolism of intact cells. Results also confirmed that underestimation of *MR* and *SRC* induced by CV inhibition is not limited to neoplastic or highly glycolytic cells. Together, these results magnify the importance of the widely overlooked recommendation [9] that *MR* (and consequently *SRC*) in intact cells be determined in the absence of CV inhibition.

## Supporting information

S1 Fig Titration of FCCP concentration in the mitochondrial stress test with untreated BMDM. (PDF)

S2 Fig Effects of inhibitor vehicles on OCR in the mitochondrial stress test with untreated BMDM. (PDF)

S3 Fig Effects of Ni^2+^ (A) and lipopolysaccharides (B) on the number of (attached) cells per well. (PDF)

S4 Fig Effects of Ni^2+^ on mitochondrial PER from BMDM during the measurement of glycolytic parameters. (PDF)

S5 Fig Effects of lipopolysaccharides on the energy metabolism of BMDM. (PDF)

## Abbreviations

2-DG: 2-deoxy-D-glucose
BMDM: bone marrow-derived macrophages
CCAC: Canadian Council on Animal Care
CCCP: carbonyl cyanide 3-chlorophenylhydrazone
CCF: CO_2_ contributing factor
CGM: complete growth medium
CV: ATP synthase, a.k.a. complex V
DAPI: 4’,6-diamidino-2-phenylindole dihydrochloride
DMEM: Dulbecco’s modified Eagle medium
DPBS: Dulbecco’s phosphate-buffered saline
ECAR: extracellular acidification rate
EFA: extracellular flux analysis
ETC: electron transport chain
FBS: fetal bovine serum
FCCP: trifluoromethoxy carbonylcyanide phenylhydrazone
LPS: lipopolysaccharides
M-CSF: macrophage colony-stimulating factor
MR: maximal respiration
OCR: oxygen consumption rate
PER: proton efflux rate
SPF: specific-pathogen-free
SRC: spare respiratory capacity.
