# Supplementary material for: ATP synthase inhibition, an overlooked confounding factor in the mitochondrial stress test
Source: PLoS One. 2025 Jul 17;20(7):e0328256. doi: 10.1371/journal.pone.0328256 (PMC12270150; doi:10.1371/journal.pone.0328256)
Supplement: S1 Fig — (PDF) [file pone.0328256.s001.pdf]

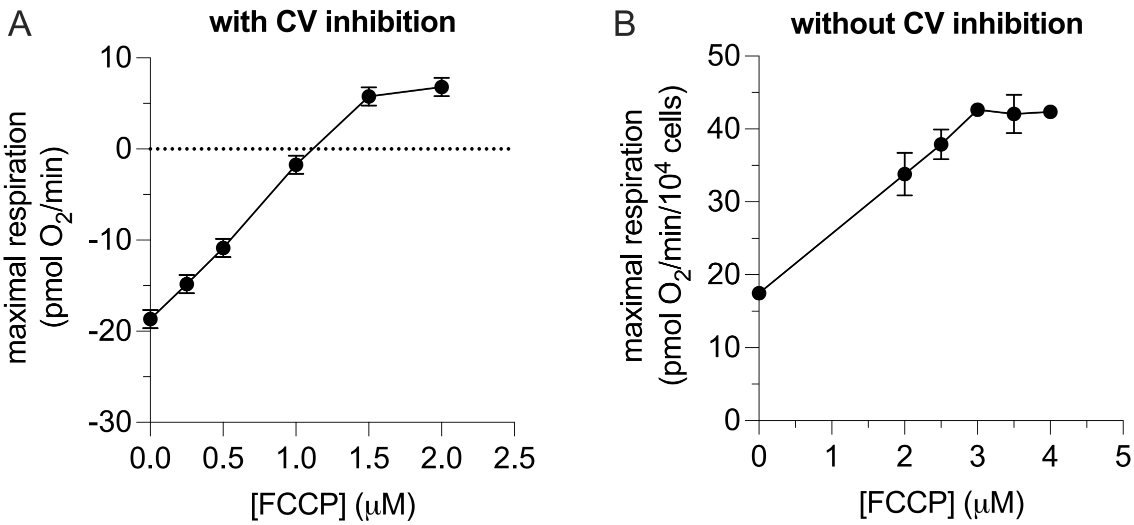

S1 Fig. Titration of FCCP concentration in the mitochondrial stress test with untreated BMDM. Murine bone marrow-derived macrophages (BMDM) were incubated for 6 h, then oxygen consumption rates (OCR) were measured by extracellular flux analysis using the mitochondrial stress test with **(A)** and without **(B)** ATP synthase (CV) inhibition with oligomycin A (1 μM). *Maximal respiration* (*MR*) measured without CV inhibition was normalized to cell number, as determined by automated microscopy, while that measured with CV inhibition was not. Data are presented as means ± SEM of sextuplicate samples of a single experiment (A) or of 2 independent experiments (each performed with sextuplicate samples) (B). SEM ≤ 0.8 pmol O_2/min/10^4 cells are covered by the symbols. FCCP: trifluoromethoxy carbonylcyanide phenylhydrazone.
