# Supplementary material for: ATP synthase inhibition, an overlooked confounding factor in the mitochondrial stress test
Source: PLoS One. 2025 Jul 17;20(7):e0328256. doi: 10.1371/journal.pone.0328256 (PMC12270150; doi:10.1371/journal.pone.0328256)
Supplement: S2 Fig — (PDF) [file pone.0328256.s002.pdf]

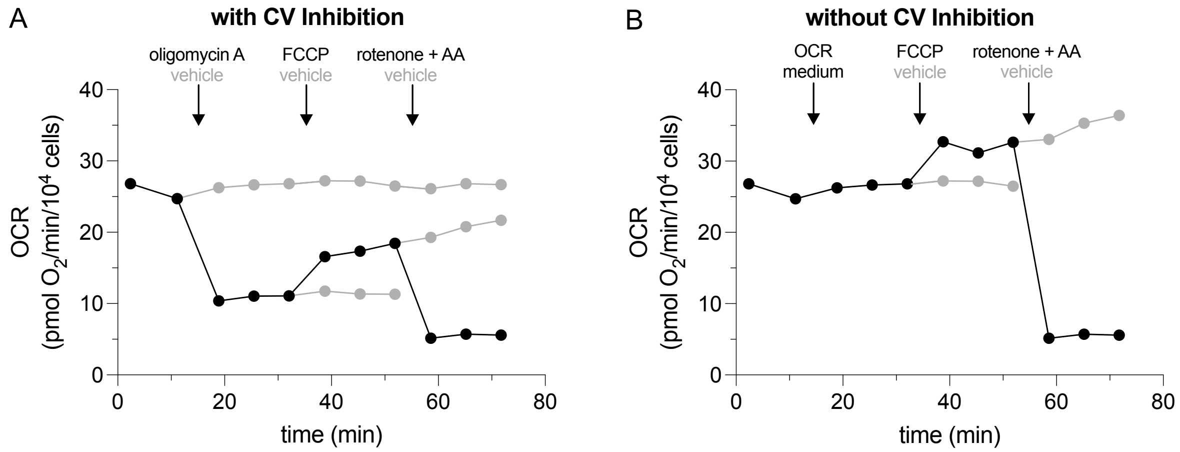

S2 Fig. Effects of inhibitor vehicles on OCR in the mitochondrial stress test with untreated BMDM. Murine bone marrow-derived macrophages (BMDM) were incubated 6 h, then oxygen consumption rates (OCR) were measured using the mitochondrial stress test with **(A)** and without **(B)** ATP synthase (CV) inhibition with oligomycin A. Vehicles (concentration of organic solvent inside the wells): oligomycin A (0.90 mM ethanol); trifluoromethoxy carbonylcyanide phenylhydrazone (FCCP) (0.63 mM [CV inhibition] or 0.95 mM [no CV inhibition] dimethyl sulfoxide [DMSO]); rotenone + antimycin A (AA) (0.70 mM DMSO and 0.24 mM ethanol). OCR measurements were normalized to cell number, as determined by automated microscopy. Data are presented as means ± SEM of sextuplicate samples. SEM ≤ 1.1 pmol O_2/min/10^4 cells are covered by the symbols. Black traces: OCR with inhibitors. Grey traces: OCR with vehicles (controls). Arrows indicate time of injection.
