# Supplementary material for: ATP synthase inhibition, an overlooked confounding factor in the mitochondrial stress test
Source: PLoS One. 2025 Jul 17;20(7):e0328256. doi: 10.1371/journal.pone.0328256 (PMC12270150; doi:10.1371/journal.pone.0328256)
Supplement: S3 Fig — (PDF) [file pone.0328256.s003.pdf]

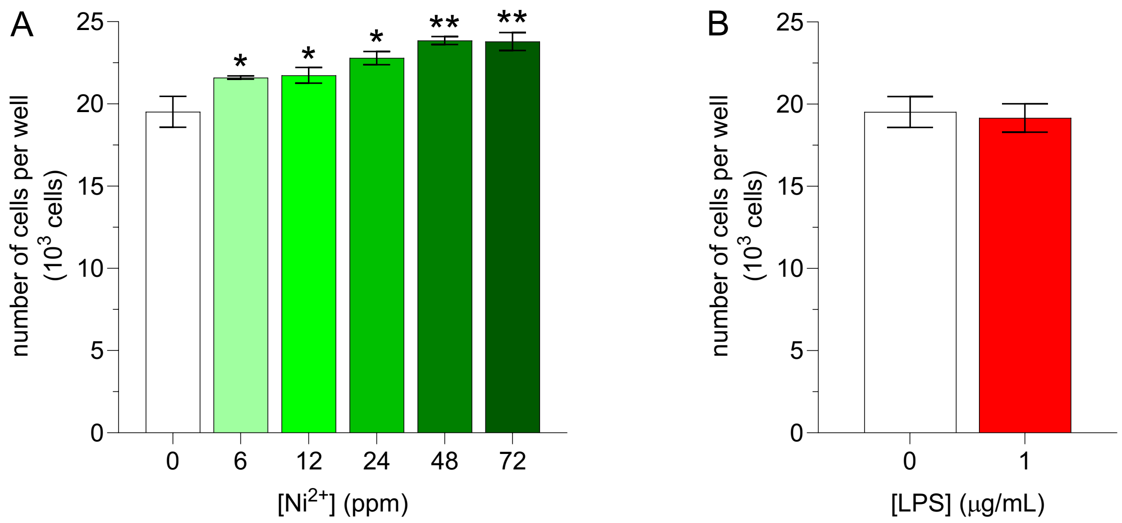

S3 Fig. Effects of Ni^{2+} (A) and lipopolysaccharides (B) on the number of (attached) cells per well. Murine bone marrow-derived macrophages (BMDM) were exposed to Ni^{2+} (0–72 ppm) or lipopolysaccharides (LPS; 0 or 1 µg/mL) for 6 h. Oxygen consumption rates (OCR) were measured by extracellular flux analysis using the mitochondrial stress test, then cells were fixed, permeabilized, stained with 4′,6-diamidino-2-phenylindole dihydrochloride (DAPI), and counted using automated microscopy, as described under *Materials and methods*. The data collected with and without CV inhibition did not differ and were therefore pooled. Note that cells attached in the divots (ca. 10% of the well bottom area) were not counted, thereby explaining, in part, the difference between the nominal number of cells seeded (32,000 per well) and the number of cells counted. Asterisks (*, **) indicate a significant difference ($p < 0.05$ and $p < 0.001$, respectively) between a given condition and its corresponding negative control (BMDM unexposed to Ni^{2+} or LPS) (one-way ANOVA followed by Dunnett's post-hoc test). Data are presented as means ± SEM of 3 independent experiments (each performed with sextuplicate samples). Note: $Ni^{2+}$and LPS were tested in parallel and thus share the same negative control.
