# Supplementary material for: ATP synthase inhibition, an overlooked confounding factor in the mitochondrial stress test
Source: PLoS One. 2025 Jul 17;20(7):e0328256. doi: 10.1371/journal.pone.0328256 (PMC12270150; doi:10.1371/journal.pone.0328256)
Supplement: S4 Fig — (PDF) [file pone.0328256.s004.pdf]

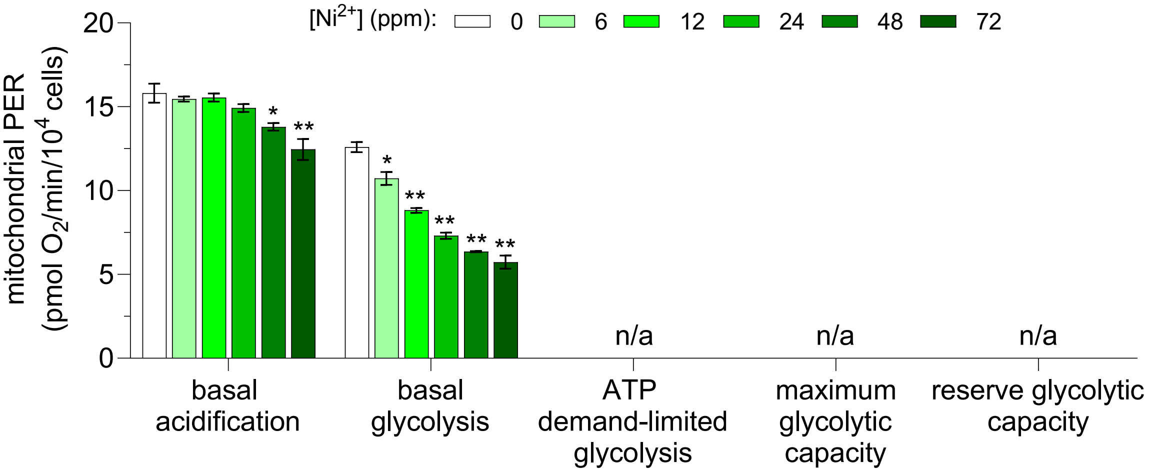

S4 Fig. Effects of Ni^{2+} on mitochondrial proton efflux rates (PER) from BMDM during the measurement of glycolytic parameters. Murine bone marrow-derived macrophages (BMDM) were exposed to Ni^{2+} (0–72 ppm) for 6 h, then mitochondrial PER were determined using the glycolysis stress test. Mitochondrial PER were calculated as described under *Materials and methods* and normalized to cell number, as determined by automated microscopy. Asterisks (*, **) indicate a significant difference ($p < 0.05$ and $p < 0.001$, respectively) between a given condition and its corresponding negative control (BMDM unexposed to Ni^{2+}) (one-way ANOVA followed by Dunnett's post-hoc test). Data are presented as means ± SEM of 3 independent experiments (each performed with sextuplicate samples). n/a: not applicable.
