# Supplementary material for: ATP synthase inhibition, an overlooked confounding factor in the mitochondrial stress test
Source: PLoS One. 2025 Jul 17;20(7):e0328256. doi: 10.1371/journal.pone.0328256 (PMC12270150; doi:10.1371/journal.pone.0328256)
Supplement: S5 Fig — (PDF) [file pone.0328256.s005.pdf]

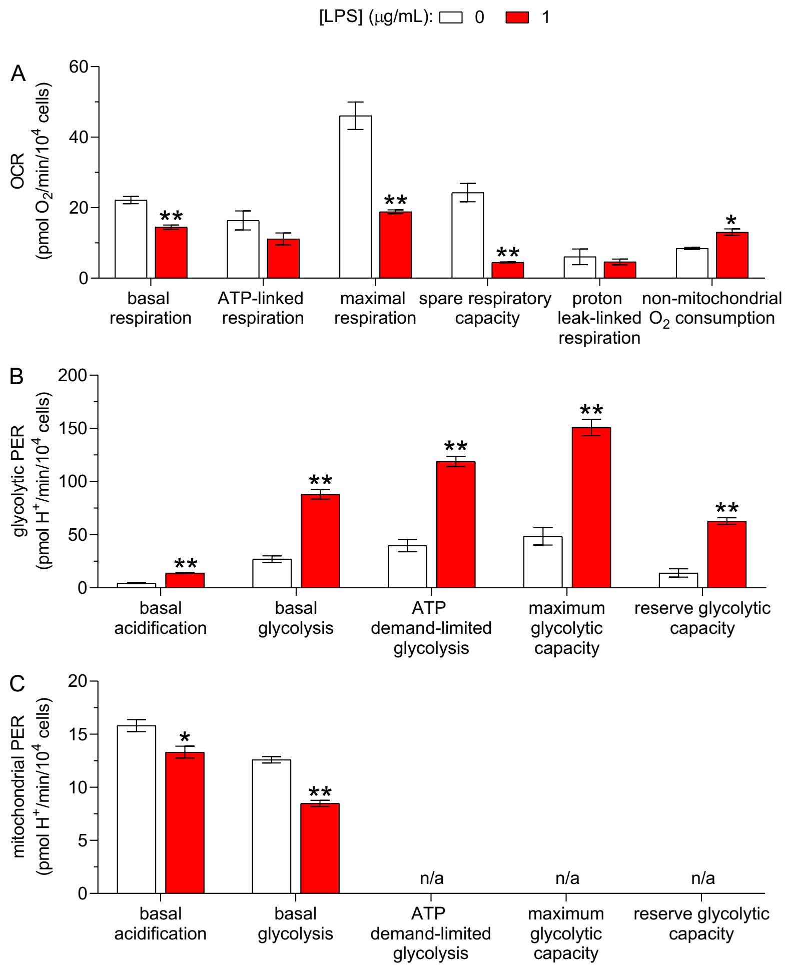

S5 Fig. Effects of lipopolysaccharides on the energy metabolism of BMDM. Murine bone marrow-derived macrophages (BMDM) were exposed to lipopolysaccharides (LPS; 0 or 1 μg/mL) for 6 h, then oxygen consumption rates (OCR) **(A)** as well as glycolytic **(B)** and mitochondrial **(C)** proton efflux rates (PER) were determined using the mitochondrial (OCR) and glycolysis stress (PER) tests, respectively. The OCR parameters were calculated using data collected with or without ATP synthase (CV) inhibition, as follows. *Basal respiration*: average of data collected with and without CV inhibition. *ATP-linked respiration* and *proton leak-linked respiration*: data collected with CV inhibition. *Maximal respiration*, *spare respiratory capacity*, and *non-mitochondrial O_2 consumption*: data collected without CV inhibition. Mitochondrial and glycolytic PER were calculated as described under *Materials and methods*. Both OCR and PER measurements were normalized to cell number, as determined by automated microscopy. Asterisks (*, **) indicate a significant difference ($p < 0.05$ and $p < 0.001$, respectively) between a given condition and its corresponding negative control (BMDM unexposed to LPS) (Student's t-tests). Data are presented as means ± SEM of 4 and 3 independent experiments for OCR and PER respectively (each performed with sextuplicate samples). Note: LPS and Ni^{2+} were tested in parallel and thus share the same negative control. n/a: not applicable.
